# Assessing the Genetic Diversity of Parents for Developing Hybrids Through Morphological and Molecular Markers in Rice (*Oryza sativa* L.)

**DOI:** 10.1186/s12284-024-00691-2

**Published:** 2024-02-24

**Authors:** Rakkimuthu Nivedha, Swaminathan Manonmani, Thiyagarajan Kalaimagal, Muthurajan Raveendran, Shanmugam Kavitha

**Affiliations:** 1 Department of Rice, Centre for Plant Breeding and Genetics, Tamil Nadu Agricultural University, Coimbatore, Tamil Nadu 641003 India; 2 Centre for Plant Breeding and Genetics, Tamil Nadu Agricultural University, Coimbatore, Tamil Nadu 641003 India; 3 Directorate of Research, Tamil Nadu Agricultural University, Coimbatore, Tamil Nadu 641003 India; 4 Department of Seed Science and Technology, Tamil Nadu Agricultural University, Coimbatore, Tamil Nadu 641003 India

**Keywords:** Genetic Diversity, Molecular Diversity, PCA, Population Structure, Cluster

## Abstract

The advancement of hybrid technology plays a crucial role in addressing yield plateau and diminishing resources in rice cultivating regions. The knowledge of genetic diversity among parental lines is a prerequisite for effective hybrid breeding program. In the current study, a set of 66 parental lines was studied for diversity based on both morphological characters and microsatellite SSR markers. The genetic variability parameters unveiled that number of productive tillers per plant, single plant yield and hundred grain weight exhibited additive gene action. Mahalanobis D^2^ statistics grouped the genotypes into ten clusters based on yield and grain traits. The principal component analysis identified four PCs with eigen value more than one accounting for 71.28% of cumulative variance. The polymorphic SSR markers produced 122 alleles among which the marker RM474 recorded the highest values for Polymorphic Information Content (0.83) and heterozygosity index (0.85). The genotypes were assembled in seven clusters based on jaccard distances using the Unweighted Pair Group method with Arithmetic Mean (UPGMA). The population structure divided the entire population into 3 subpopulations. In both clustering, there was difference in the assembling of genotypes, but, good performing genotypes identified through PCA were positioned in different clusters in both approaches. The genotypes CBSN 495 and CBSN 494 located in different clusters were identified as the potential restorers for high yielding and short duration hybrids. The hybridization among CRR Dhan 310, CRR Dhan 315, IR64 DRT, CB 17135 and WGL 347 can be performed to develop climate smart varieties with improved nutrition.

**Supplementary Information:**

The online version contains supplementary material available at 10.1186/s12284-024-00691-2.

## Background

Rice, filling the calorie requirement of half of the world population faces a challenge marked by yield plateau in the recent years. This challenge compounded with escalating population necessitates urgent focus on increasing productivity which in turn is achieved by developing high yielding hybrids. The success in hybrid breeding largely depends on the genetic diversity of parents used for crossing. This emphasizes the scope of examining genetic diversity among various rice genotypes to help in the selection of precise parents for hybrid breeding. The literatures provide many studies of diversity using both morphological and molecular clustering such as Singh et al. (2022) for 47 rice genotypes, Pathak et al. (2020) for 29 local rice cultivars, Islam et al. (2019) in 28 restorer lines, Vengadessan et al. (2016) for 33 traditional and 12 improved rice cultivars and Rahman et al. (2011) for 21 rice varieties. Hence, the present study was conducted to assess the genetic diversity of 66 rice parental lines using morphological and molecular clustering for the development of hybrids.

## Introduction

Rice stands as a global significant crop nourishing more than 50% of the world’s population (Muthayya et al. 2014). With the demographic expansion under ever declining resources, there is an urgent need to harness heterosis in crop plants. In case of rice, a highly self-pollinated crop, the study of genetic diversity assumes a critical role in the selection of diverse parents aimed for attaining maximum heterosis and transgressive segregants in the successive generations. The understanding of diversity among genotypes is achieved through various multivariate statistical analysis. Mahalanobis D^2^ statistics is a potent statistical tool to quantify the genetic distance between genotypes based on replicated data of multiple variables (Mahalanobis 1936). The Principal Component Analysis helps in streamlining the selection process by condensing the total number of variables into key variables with major contribution to the total variation (Devasena et al. 2023; Sheela et al. 2020). The advent of molecular markers enabled the characterization of genotypes more precisely at DNA level differences free from the interference of environmental interactions. The microsatellite Simple Sequence Repeat markers exhibit great level of allelic polymorphism and has widespread application in rice for various molecular studies (Rahman et al. 2011). Further the resolution of population into distinct subgroups is made possible through structure analysis. Assessing the genetic diversity among genotypes based on molecular markers ensure enhanced resolution and significant time savings compared to traditional morphological study. In this regard, the present study was undertaken to assess the genotypic diversity among 66 rice parental lines, employing both morphological and molecular based clustering. The findings aim to provide valuable insights into the significance of these diverse genotypes in the context of rice breeding, offering opportunities for the development of high yielding hybrids to meet the ever-growing global demand.

## Materials and Methods

A total of 66 parental lines including released varieties from Tamil Nadu, Andhra Pradesh, Telangana, improved varieties for nutrition and abiotic stress tolerance, *indica-japonica* cross derivatives in which *indica* lines were confirmed for restorer genes and wild rice Multi-parent advanced generation inter-cross (MAGIC) derivatives were evaluated at Paddy Breeding Station, Tamil Nadu Agricultural University, India. The station is located at 11^0^N latitude and 77^0^E longitude with an elevation of 426.72 meters above the sea level. The experiment was carried out in *Rabi*, 2022 in Randomized Block Design with three replications. The list of genotypes with their geographical origin and subspecies type are presented in Table 1. The 30 days old seedlings were transplanted with a spacing of 20 × 20 cm. The fertilizer application and intercultural operations were carried out as per the recommended standard. The observations for ten agronomical and grain traits viz., days to 50% flowering (DFF), plant height (PH), number of productive tillers per plant (NPTP), panicle length (PL), flag leaf length (FLL), single plant yield (SPY), hundred grain weigh (HGW), grain length (GL), grain breadth (GB), grain L/B ratio (L/B) were randomly recorded in 5 plants of each genotype in each replication. The mean values of the genotypes for all the recorded traits are given in Table S1.

**Table 1:** The list of genotypes with their geographical origin and subspecies type

S No	Genotype name	Geographical Origin	Subspecies
1	CO 51	Tamil Nadu	*indica*
2	AD 12132	Tamil Nadu	*indica*
3	CO 52	Tamil Nadu	*indica*
4	CO55	Tamil Nadu	*indica*
5	ADT 53	Tamil Nadu	*indica*
6	WGL 283	Telangana	*indica*
7	ADT 56	Tamil Nadu	*indica*
8	TRY3	Tamil Nadu	*indica*
9	CO43 Sub 1	Tamil Nadu	*indica*
10	CR 1009 Sub 1	Odisha	*indica*
11	CO 54	Tamil Nadu	*indica*
12	CRR Dhan 315	Odisha	*indica*
13	TKM 13	Tamil Nadu	*indica*
14	White Ponni mutant	Tamil Nadu	*indica*
15	CBSN 494	Tamil Nadu	*indica-japonica* cross derivative
16	RNR 15048	Telangana	*indica*
17	AD 18073	Tamil Nadu	*indica*
18	CRR Dhan 310	Odisha	*indica*
19	AD 13253	Tamil Nadu	*indica*
20	MTU 1121	Andhra Pradesh	*indica*
21	MTU 1156	Andhra Pradesh	*indica*
22	DRR Dhan 40	Andhra Pradesh	*indica*
23	MTU 1210	Andhra Pradesh	*indica*
24	WGL 347	Telangana	*indica*
25	WGL 21356	Telangana	*indica*
26	CBSN 495	Tamil Nadu	*indica-japonica* cross derivative
27	WGL 739	Telangana	*indica*
28	WGL 3962	Telangana	*indica*
29	CBSN 496	Tamil Nadu	*indica-japonica* cross derivative
30	WGL 32100	Telangana	*indica*
31	CB 19127	Tamil Nadu	*indica*
32	CBSN 497	Tamil Nadu	Wild rice derivative
33	CB 17135	Tamil Nadu	*indica*
34	CB 20165	Tamil Nadu	*indica*
35	CB 20142	Tamil Nadu	*indica*
36	CO 51 Pyr A10	Tamil Nadu	*indica*
37	CBSN 498	Tamil Nadu	*indica-japonica* cross derivative
38	MTU 1155	Andhra Pradesh	*indica*
39	CBSN 499	Tamil Nadu	*indica-japonica* cross derivative
40	CBSN 500	Tamil Nadu	*indica-japonica* cross derivative
41	CO 51 Pyr A1	Tamil Nadu	*indica*
42	CBSN 501	Tamil Nadu	*indica-japonica* cross derivative
43	MTU 1153	Andhra Pradesh	*indica*
44	CBSN 502	Tamil Nadu	*indica-japonica* cross derivative
45	CB 19126	Tamil Nadu	*indica*
46	CBSN 503	Tamil Nadu	*indica-japonica* cross derivative
47	CBSN 504	Tamil Nadu	*indica-japonica* cross derivative
48	CBSN 505	Tamil Nadu	*indica-japonica* cross derivative
49	CBSN 506	Tamil Nadu	*indica-japonica* cross derivative
50	CBSN 507	Tamil Nadu	*indica-japonica* cross derivative
51	CBSN 508	Tamil Nadu	*indica-japonica* cross derivative
52	CBSN 509	Tamil Nadu	Wild rice derivatives
53	CBSN 510	Tamil Nadu	*indica*
54	CBSN 511	Tamil Nadu	Wild rice derivative
55	IR 64 DRT	Andhra Pradesh	*indica*
56	CBSN 512	Tamil Nadu	*indica*
57	CBSN 513	Tamil Nadu	*indica-japonica* cross derivative
58	CBSN 514	Tamil Nadu	*indica*
59	CBSN 515	Tamil Nadu	*indica-japonica* cross derivative
60	CBSN 516	Tamil Nadu	*indica*
61	CO 51 Pyr A7	Tamil Nadu	*indica*
62	CBSN 517	Tamil Nadu	*indica-japonica* cross derivative
63	CBSN 518	Tamil Nadu	*indica-japonica* cross derivative
64	CBSN 519	Tamil Nadu	*indica-japonica* cross derivative
65	CO 53	Tamil Nadu	*indica*
66	CBSN 520	Tamil Nadu	Wild rice derivative

### Molecular Assay

The Genomic DNA was extracted from young leaf samples of 3–4 weeks old seedlings following Doyle and Doyle’s protocol (1987). The Polymerase Chain Reaction (PCR) was performed in a 10 μl reaction mixture: Template DNA 2 μl (20ng/μl), forward primer 0.5 μl (10μM), reverse primer 0.5 μl (10μM), master mix 4 μl (2X) and sterile water (3 μl) for 51 SSR primers distributed across all the chromosomes. The temperature profile used for PCR was initial denaturation at 95 °C (5 min) followed by 35 cycles of denaturation at 94 °C (1 min), annealing at 55 °C (45 s), extension at 72 °C (30 s) and a final extension at 72 °C (10 min). The samples were then held at 4 °C until retrieval. The resulting amplified products were separated on a 3% polyacrylamide gel in 1X TBE buffer alongside a 100 bp ladder (Bio-Helix), visualized under UV transillumination by Bio-Rad imaging system. The details of polymorphic markers along with range of amplified base pair are given in Table 2. The 30 polymorphic primers were scored as ‘1’ and ‘0’ for the presence or absence of alleles respectively in all the 66 genotypes. The Jaccard distance based molecular cluster analysis was performed using this scoring while base pair scoring was applied for structure analysis.

**Table 2:** The list of polymorphic markers along with their sequence, chromosome number, annealing temperature and range of amplified product size

S.No.	Marker	Sequence (5’-3’)	Chromosome number	Annealing Temperature	Amplified product size (bp)
1	RM1	F: GCGAAAACACAATGCAAAAA R: GCGTTGGTTGGACCTGAC	1	55	90–115
2	RM31	F: CGCTCCTCCACTCTTCTCCTACC R: CGTGCAGAAAGTCCATTACTCTCC	5	55	140–180
3	RM44	F: ACGGGCAATCCGAACAACC R: CGAGGATGGTTGTTCACTTG	8	55	99–105
4	RM205	F: CTGGTTCTGTATGGGAGCAG R: CTGGCCCTTCACGTTTCAGTG	9	55	230–250
5	RM208	F: TCTGCAAGCCTTGTCTGATG R: TAAGTCGATCATTGTGTGGACC	2	55	173–290
6	RM216	F: GCATGGCCGATGGTAAAG R: GGATTTTCTGATAGCGGTAA	10	55	140–160
7	RM228	F: CTGGCCATTAGTCCTTGG R: GCTTGCGGCTCTGCTTAC	10	55	100–160
8	RM232	F: CCGGTATCCTTCGATATTGC R: CCGACTTTTCCTCCTGACG	3	55	158–190
9	RM258	F: TGCTGTATGTAGCTCGCACC R: TGGCCTTTAAAGCTGTCGC	10	55	150–290
10	RM267	F: TGCAGACATAGAGAAGGAAGTG R: AGCAACAGCACAACTTGATG	5	55	156–175
11	RM313	F: GAGGTACTTCCTCCGTTTCAC R: AGTCAGCTCACTGTGCAGTG	1	55	111–160
12	RM315	F: GAGGTACTTCCTCCGTTTCAC R: AGTCAGCTCACTGTGCAGTG	1	55	105–140
13	RM443	F: GATGGTTTTCATCGGCTACG R: AGTCCCAGAATGTCGTTTCG	1	55	100–124
14	RM445	F: CGTAACATGCATATCACGCC R: ATATGCCGATATGCGTAGCC	7	55	200–295
15	RM461	F: GGACATACGTACAGTGGTTGATGC R: GTGCATCAAACGACAAACTCTCC	6	55	190–200
16	RM471	F: ACGCACAAGCAGATGATGAG R: GGGAGAAGACGAATGTTTGC	4	55	100–106
17	RM474	F: AAGATGTACGGGTGGCATTC R: TATGAGCTGGTGAGCAATGG	10	55	205–275
18	RM481	F: TAGCTAGCCGATTGAATGGC R: TAGCTAGCCGATTGAATGGC	7	55	150–197
19	RM514	F: AGATTGATCTCCCATTCCCC R: CACGAGCATATTACTAGTGG	3	55	220–280
20	RM555	F: TTGGATCAGCCAAAGGAGAC R: CCTGTACGTTGATCCGAAGC	2	55	215–223
21	RMS-SF21 − 5	F: GAGTTGGGGGTCGAGAAATC R: CGTACGTGCGGCTAGGATCAA	1	55	135–200
22	RM1108	F: GCTCGCGAATCAATCCAC R: GCTGGATCACAGATCATTGC	10	55	124–142
23	RM3317	F: AGCAACCTGACAGAAGAATG R: TCTCGTTGAGTTGGAAGAAG	4	55	155–180
24	RM3530	F: GTAGATCCGGTCAGCTCCTC R: CAAGGAGATTCCCTTCCATG	1	55	160–195
25	RM5359	F: CGTGATCTCGTGCATCCC R: CCCTCAGGAGCTTCATGAAC	1	55	175–200
26	RM6344	F: ACACGCCATGGATGATGAC R: TGGCATCATCACTTCCTCAC	7	50	100–120
27	RM6869	F: GAGCTCCTTGTAGTGACCCG R: ATCAGCCTCGCCAGCTTC	12	61	126–140
28	RM10318	F: TGTCTCACACATTGCACACTTACC R: GGCCTAACCCAACACATGTCC	1	55	175–196
29	RMS-PPR9 − 1	F: GAGTTTTGAATAGATTTACGTGTGGA R: AGTGTCCAGATTCGTAGTAATGC	10	58	114–168
30	DRRMRf3 10	F: GATGGCAAGCTTCAGAACA R: CTAATTCTGGGCGAGCAAAG	1	55	210–250

### Statistical Analysis

The Genetic variability parameters, Analysis of Variance and Principal Component Analysis were performed using the packages ‘variability’, ‘Agricolae’, ‘FactoMineR’ and ‘factoextra’ of R studio 4.2.3. The Mahalanobis D^2^ statistics and clustering by Tocher’s method was done in TNAUSTAT software (Manivannan 2014). The marker scorings were analysed with R-shiny based package ‘PBPERFECT’ (Allan 2023) for Jaccard distance, molecular cluster, PIC value and Heterozygosity index. The structure analysis was carried out using STRUCTURE 2.3.4 software and the results were viewed in STRUCTURE HARVESTER. The Analysis of Molecular Variance (AMOVA), genetic differentiation (Fst), genetic diversity parameters viz., observed heterozygosity (Ho) and expected heterozygosity (He) were analysed using GenAlex 6.5 (Peakall and Smouse 2007).

## Results

### Genetic Variability

**Fig. 1:**
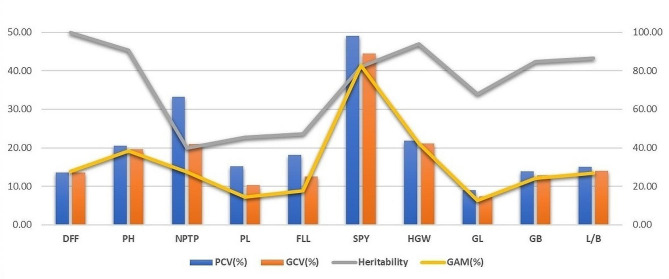
Genetic parameters for the yield and grain traits PCV -Phenotypic Co-efficient of Variation; GCV – Genotypic Co-efficient of Variation; GAM – Genetic Advance as per cent of Mean DFF – Days to 50 % flowering; PH – Plant height (cm); NPTP – Number of productive tillers per plant; PL – Panicle length (cm); FLL – Flag leaf length (cm); SPY – Single plant yield (g); HGW – Hundred grain weight (g); GL – Grain length (cm); GB – Grain breadth (cm); L/B – Grain Length Breadth ratio

The Phenotypic Co-efficient of Variation (PCV), Genotypic Co-efficient of Variation (GCV), broad sense heritability and Genetic Advance as per cent of Mean (GAM) were calculated for all the traits under study. The estimates of PCV were more than GCV for all the traits indicating the influence of environment in the expression of the traits (Fig. 1). High PCV and GCV were observed for number of productive tillers per plant (33.30, 21.01), single plant yield (49.03, 44.40) and hundred grain weight (21.79, 21.13). All other traits except grain length exhibited low range for both PCV and GCV. The range of heritability and GAM was derived between moderate to high for which the traits viz., days to 50% flowering, plant height, number of productive tillers per plant, single plant yield, hundred grain weight, grain breadth and grain length breadth ratio had high heritability coupled with high GAM. These traits offer advantage by responding to selection and therefore can be taken as a criterion for selection of parents.

### Morphological Diversity

**Fig. 2:**
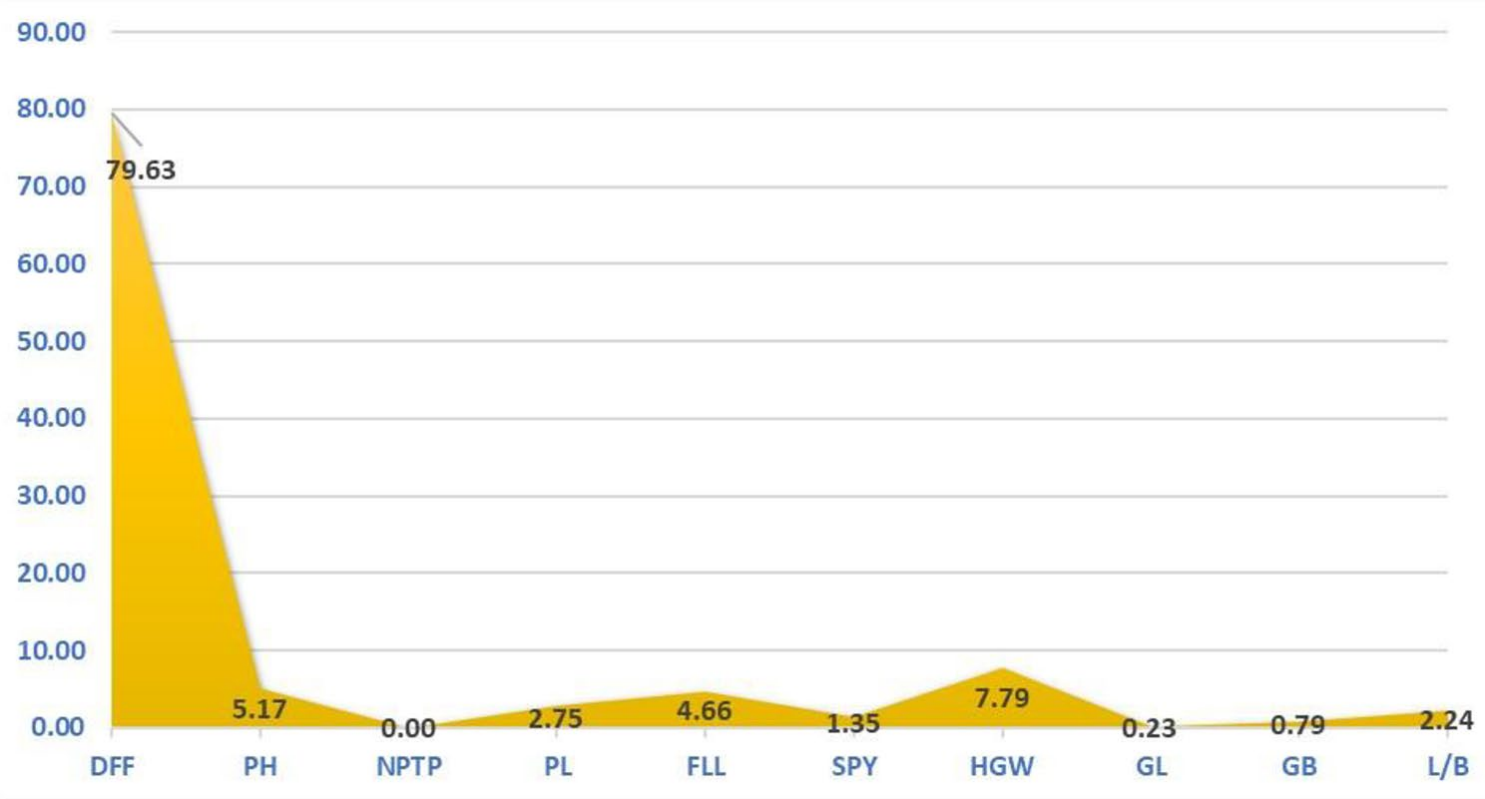
The individual contribution of traits to the total divergence DFF – Days to 50 % flowering; PH – Plant height (cm); NPTP – Number of productive tillers per plant; PL – Panicle length (cm); FLL – Flag leaf length (cm); SPY – Single plant yield (g); HGW – Hundred grain weight (g); GL – Grain length (cm); GB – Grain breadth (cm); L/B – Grain Length Breadth ratio

The Analysis of Variance (ANOVA) for the ten biometrical traits revealed significant variation among all the genotypes (Table 3). The genetic diversity of parental lines was estimated by Mahalanobis D^2^ statistics and the genotypes were categorized into clusters by Tocher’s method. All the 66 genotypes were grouped into ten clusters based on D^2^ values in a random manner without any consideration of origin or subspecies (Table 4). The cluster I was the largest comprising 19 genotypes followed by cluster IV (10 genotypes). The clusters V, III, II and VI contained 9, 7, 6 and 2 genotypes respectively. The clusters VII, VIII and X contained 4 genotypes in each and cluster IX was identified as a solitary cluster with only one genotype viz., CBSN 495. The intra and inter cluster distances (Table 5) portray the diversity within and among the different clusters respectively. The maximum inter cluster distance was recorded between clusters III and X (5952.26) followed by clusters VIII and X (4915.97) and clusters IX and X (4259.24). The cluster X comprised of wild rice magic derivatives therefore it was showing maximum diversity with most of the other clusters. The minimum inter cluster distance was observed between clusters I and IV (197.00) followed by clusters II and VI (215.24) emphasizing close relation between them. The maximum intra cluster distance was observed for cluster VII (175.97) followed by cluster X (158.30) and cluster IV (153.66). The minimum intra cluster distance was noticed for cluster IX (0) since it was solitary with single genotype followed by cluster III (102.85). The trait days to 50% flowering (79.63%) exerted maximum contribution towards total divergence (Table S2 and Fig. 2). It was followed by hundred grain weight (7.79%), plant height (5.17%), flag leaf length (4.66%) and panicle length (2.75%). This showed that the genotypes had a wide variation with regard to duration. The cluster mean for all the traits are given in Table 6. The cluster III recorded low mean values for days to 50% flowering (79.05) and plant height (68.52). The high mean values were registered for panicle length (25.50) and single plant yield (28.63) by cluster VII, flag leaf length (32.37) by cluster VI, hundred grain weight (2.57) and plant height (118.33) by cluster IX. The cluster II had comparably high mean values for number of productive tillers per plant, panicle length, flag leaf length and low mean value for grain breadth. The genotypes from this cluster shall be employed as parents for producing superior hybrids with medium slender grain type.

**Table 3:** ANOVA for all the biometrical traits

Source of variation	df	DFF	PH	NPTP	PL	FLL	SPY	HGW	GL	GB	L/B
Genotype	65	514.678^**^	786.012^**^	67.142^**^	29.576^**^	52.621^**^	250.260^**^	0.409^**^	0.012^**^	0.004^**^	0.595^**^
Replication	2	0.384	8.318	1.702	4.073	12.117	0.250	0.014	0.000	0.000	0.018
Error	130	0.409	25.739	22.492	1.335	14.311	17.043	0.009	0.002	0.000	0.030

DFF – Days to 50% flowering; PH – Plant height (cm); NPTP – Number of productive tillers per plant; PL – Panicle length (cm); FLL – Flag leaf length (cm); SPY – Single plant yield (g); HGW – Hundred grain weight (g); GL – Grain length (cm); GB – Grain breadth (cm); L/B – Grain Length Breadth ratio

**Table 4:** Clustering of genotypes by Tocher’s method

Cluster	No of genotypes	Name of genotypes
I	19	CO 51, AD 12132, ADT 53, ADT 56, White Ponni mutant, TKM 13, WGL 32100, CB 20165, CBSN 508, MTU 1121, MTU 1210, WGL 739, MTU 1153, CB 19126, CBSN 504, DRR Dhan 40, CB 19127, CB 20142, CBSN 517
II	6	CO 52, CO55, CO 54, RNR 15048, AD 13253, CBSN 513
III	7	WGL 283, WGL 347, WGL 3962, CBSN 498, CBSN 499, CBSN 518, CBSN 519
IV	10	TRY3, CRR Dhan 310, MTU 1156, CBSN 496, CBSN 506, CBSN 507, CBSN 510, CBSN514, CBSN 515, CO 53
V	9	CO43 Sub 1, CR 1009 Sub 1, AD 18073, CBSN 497, CBSN 502, CBSN 503, CBSN 505, CBSN 509, IR 64 DRT
VI	2	CRR Dhan 315, CBSN 501
VII	4	CBSN 494, CB 17135, CO 51 Pyr A10, CBSN 500
VIII	4	WGL 21356, MTU 1155, CO 51 Pyr A1, CO 51 Pyr A7
IX	1	CBSN 495
X	4	CBSN 511, CBSN 512, CBSN 516, CBSN 520

**Table 5:** The inter and intra cluster distances among ten clusters

Cluster	I	II	III	IV	V	VI	VII	VIII	IX	X
I	**105.64**	393.47	636.94	197.00	1636.81	725.04	247.71	344.96	451.92	3047.08
II		**141.46**	1673.20	363.45	667.10	215.24	800.47	1141.26	1079.23	1591.66
III			**102.85**	839.62	3896.65	2289.26	362.01	221.77	382.03	5952.26
IV				**153.66**	1401.05	568.02	329.09	557.70	370.14	2677.54
V					**127.55**	351.05	2469.32	3052.04	2658.64	358.67
VI						**147.93**	1238.71	1731.40	1345.63	995.07
VII							**175.97**	236.19	267.92	4124.15
VIII								**146.04**	420.38	4915.97
IX									**0.00**	4259.24
X										**158.30**

Numbers in bold font – intra cluster distances; numbers in normal font – inter cluster distances

**Table 6:** The cluster mean for all the biometrical traits

Cluster	DFF	PH	NPTP	PL	FLL	SPY	HGW	GL	GB	L/B
1	92.86	72.67	19.18	20.69	28.81	22.13	1.54	0.77	0.24	3.23
2	102.56	81.22	22.50	25.00	31.26	23.39	1.53	0.78	0.23	3.39
3	79.05	68.52	17.38	21.57	27.19	17.79	1.96	0.88	0.26	3.34
4	93.73	92.00	16.93	25.00	30.12	18.05	1.92	0.79	0.29	2.73
5	115.96	79.89	17.52	22.00	28.43	18.11	1.80	0.78	0.28	2.82
6	106.67	97.67	18.50	23.50	32.37	25.67	2.04	0.87	0.26	3.36
7	86.42	98.67	17.75	25.50	25.33	28.63	1.65	0.81	0.25	3.28
8	84.00	68.92	18.33	18.75	22.88	13.50	1.34	0.75	0.25	3.13
9	84.67	118.33	11.67	24.00	26.33	9.50	2.57	0.92	0.32	2.88
10	124.75	96.83	17.67	21.25	29.50	13.13	2.07	0.78	0.27	3.01

DFF – Days to 50 % flowering; PH – Plant height (cm); NPTP – Number of productive tillers per plant; PL – Panicle length (cm); FLL – Flag leaf length (cm); SPY – Single plant yield (g); HGW – Hundred grain weight (g); GL – Grain length (cm); GB – Grain breadth (cm); L/B - Grain Length Breadth ratio

### Principal Component Analysis

**Fig. 3:**
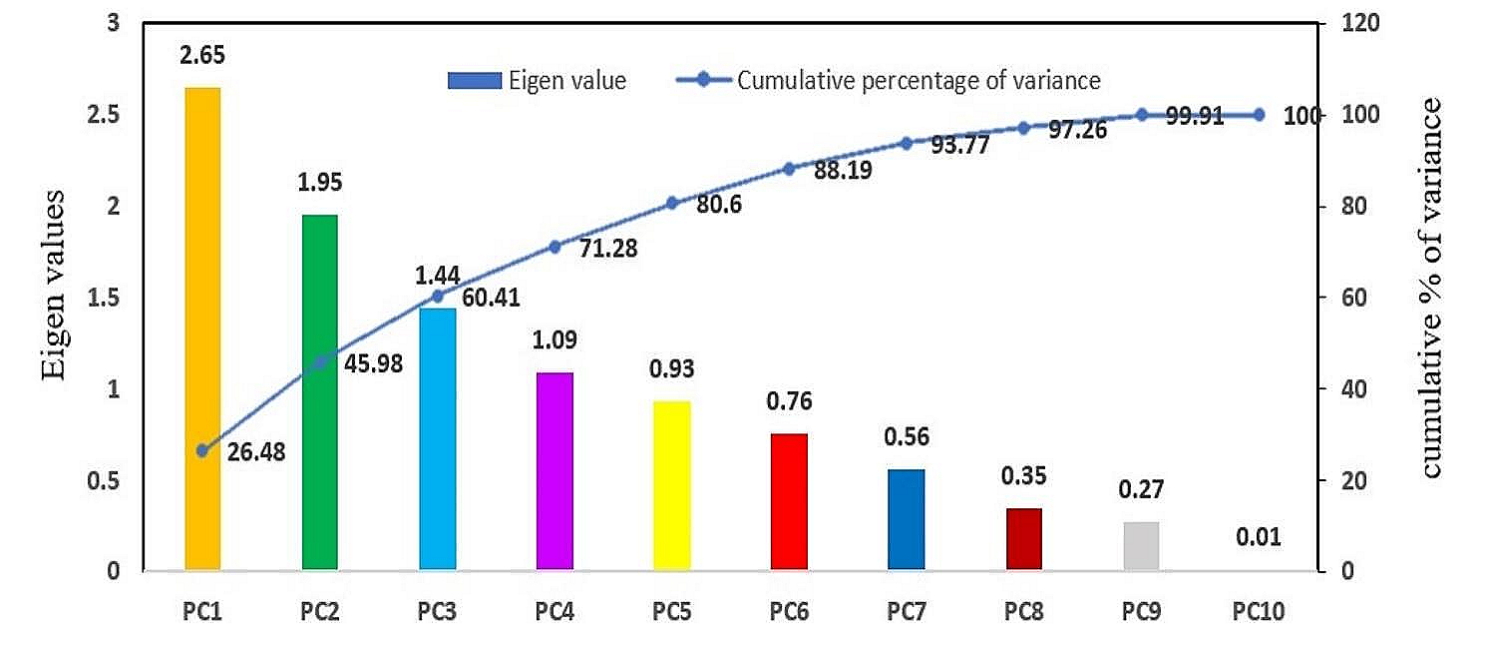
Scree plot depicting the relation between all the principal components and the contribution of variation percent

The PCA reduces the dimensionality of data by identifying the most prominent few variables responsible for variation in the genotypes. The principal component analysis for the morphological traits under study revealed the presence of variability among all the parental lines. The factor loadings of each variable, eigen values, percent of variance and cumulative percent of variance for all the ten principal components are given in Table 7. The first four PCs had eigen value > 1 and accounted for a cumulative variance of 71.28%. PC1 (eigen value of 2.65) contributed 26.48% of total variance followed by PC2 (19.50%), PC3 (14.43%) and PC4 (10.87%). The remaining PCs altogether contributed 28.72% to the total divergence of the genotypes. Days to 50% flowering (0.205), plant height (0.423), panicle length (0.322), flag leaf length (0.247), hundred grain weight (0.425), grain length (0.152) and grain breadth (0.501) exhibited positive weightage to PC axis 1. The PC2 showed positive loadings for days to 50% flowering (0.186) and grain breadth (0.256). In PC3, hundred grain weight (0.321), grain length (0.390) and grain breadth (0.208) and in PC4, days to 50% flowering (0.557) and hundred grain weight (0.213) had positive weightage to the corresponding PC axis respectively. The scree plot (Fig. 3) displaying the relation between all the principal components and the contribution of variation percent depicted the PC1’s dominant role in variation among the genotypes guiding trait selection to harness maximum variability.

**Table 7:** The factor loadings, eigen values, percent of variance and cumulative percent of variance for all principal components

Variables	PC1	PC2	PC3	PC4	PC5	PC6	PC7	PC8	PC9	PC10
DFF	0.205	0.186	-0.339	0.557	-0.130	-0.578	0.019	0.355	-0.145	-0.019
PH	0.423	-0.222	-0.128	-0.056	-0.459	-0.118	0.278	-0.631	-0.227	-0.023
NPTP	-0.129	-0.287	-0.244	0.449	0.638	0.183	0.297	-0.275	-0.186	-0.010
PL	0.322	-0.361	-0.259	-0.031	-0.187	0.458	0.359	0.539	0.185	0.009
FLL	0.247	-0.073	-0.576	-0.100	0.104	0.186	-0.730	-0.116	0.003	-0.004
SPY	-0.052	-0.168	-0.334	-0.626	0.328	-0.526	0.250	0.078	0.110	0.019
HGW	0.425	-0.224	0.321	0.213	0.218	-0.219	-0.115	-0.140	0.702	-0.001
GL	0.152	-0.537	0.390	-0.064	0.104	-0.153	-0.258	0.247	-0.496	-0.353
GB	0.501	0.256	0.208	-0.128	0.305	0.064	0.037	0.093	-0.328	0.639
L/B	-0.374	-0.515	-0.001	0.125	-0.247	-0.148	-0.170	0.016	0.032	0.682
Eigen value	2.65	1.95	1.44	1.01	0.93	0.76	0.56	0.35	0.27	0.01
% variance	26.48	19.50	14.43	10.87	9.33	7.59	5.58	3.49	2.65	0.10
cumulative % of variance	26.48	45.977	60.405	71.277	80.603	88.19	93.77	97.255	99.906	100

**Fig. 4:**
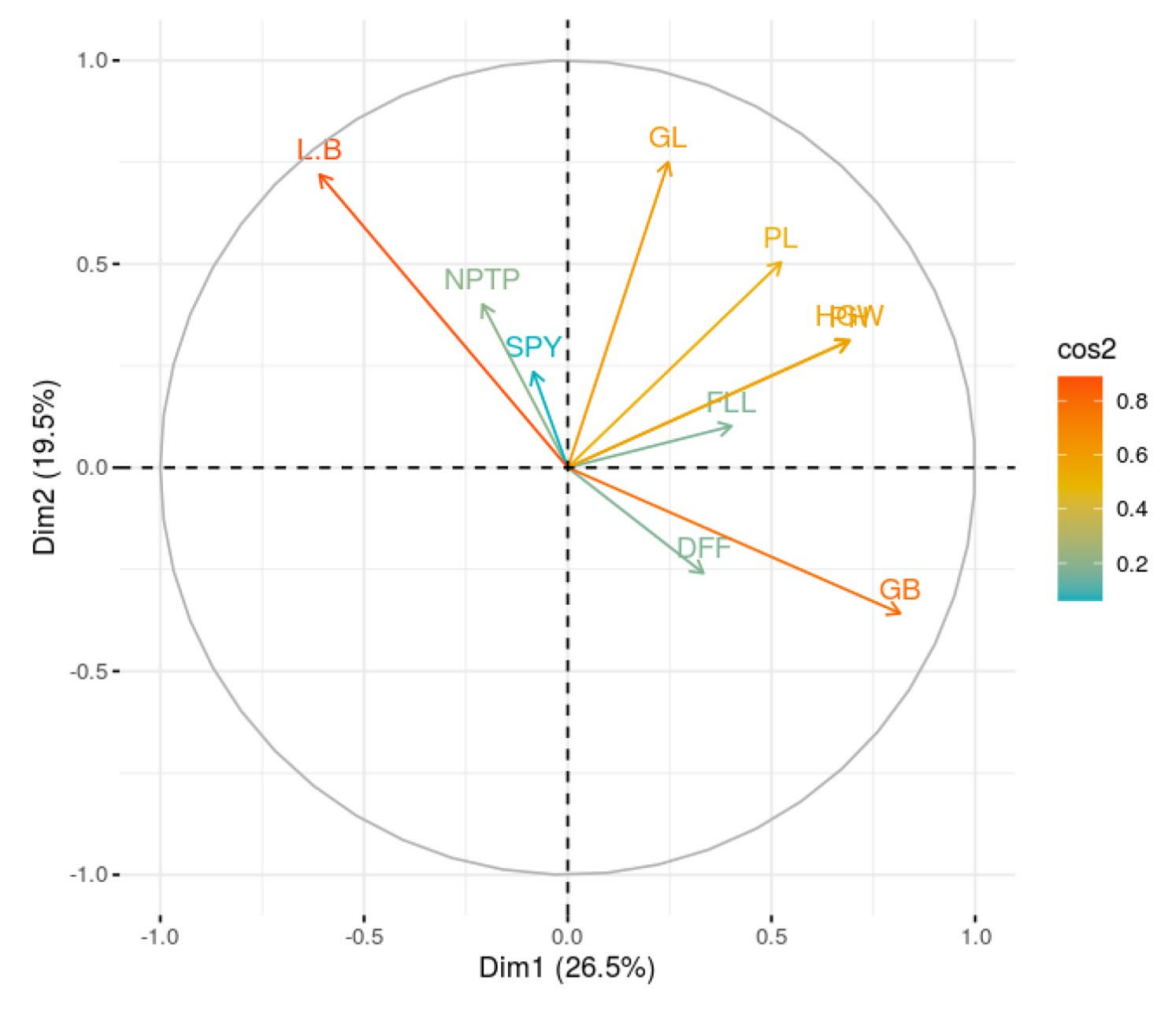
PCA for variables DFF – Days to 50% flowering; PH – Plant height (cm); NPTP – Number of productive tillers per plant; PL – Panicle length (cm); FLL – Flag leaf length (cm); SPY – Single plant yield (g); HGW – Hundred grain weight (g); GL – Grain length (cm); GB – Grain breadth (cm); L/B – Grain Length Breadth ratio

**Fig. 5:**
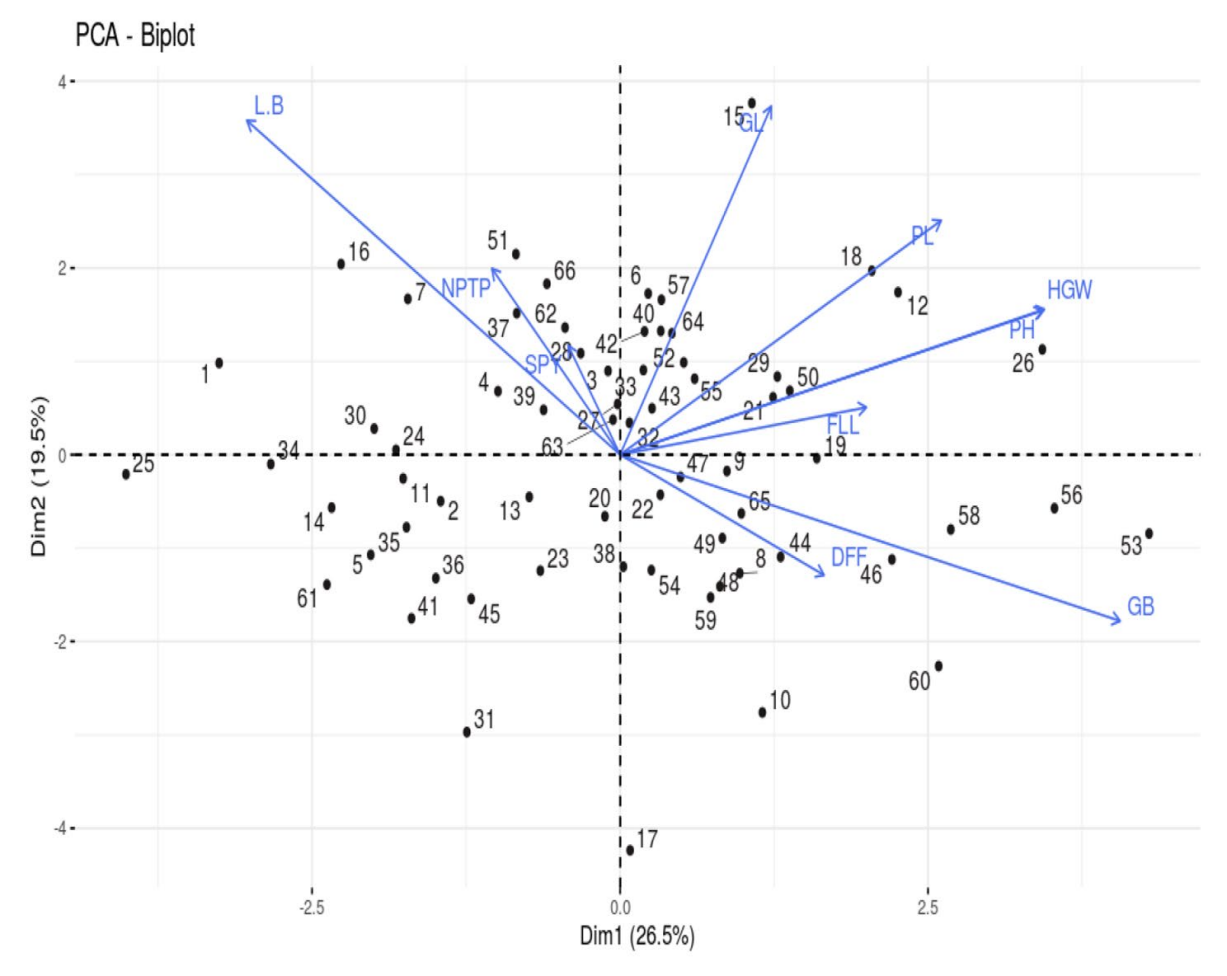
PCA Biplot The numbers denote genotypes* and the vectors correspond to the biometrical traits The genotypes are numbered according to the list provided in Table 1

The degree and the relation between variables are shown as vectors in PCA plot for variables (Fig. 4). The length of the variable vector is directly proportional to the contribution of respective trait to the total divergence. Considering the traits with positive loadings to the first four PCs, the longest vector is observed for grain breadth followed by plant height, hundred grain weight, grain length and panicle length. The maximum contribution by the traits to the total divergence is pronounced in the above-mentioned order of vector lengths. Further, angle between the vectors determine the direction of association among the variables. If the angle between the vectors is acute (< 90^0^) or obtuse (> 90^0^), there exist a positive or negative correlation between the corresponding traits respectively. If the vectors of two traits are at right angle (90^0^) to each other, they are said to be uncorrelated (Christina et al. 2021). The positively correlated variables with single plant yield were number of productive tillers per plant, grain length, panicle length, plant height, hundred grain weight and grain length breadth ratio. The variable vectors viz., flag leaf length, grain breadth and days to 50% flowering produced obtuse angle with vector of single plant yield showing negative association. The interaction between genotypes and the variables are depicted in PCA biplot (Fig. 5). The genotypes located around the variable vectors in the same quadrant are meant to be best performers for the particular trait. The genotypes viz., CBSN 495, CRR Dhan 315, CBSN 494, CRR Dhan 310, AD 13253, MTU 1156 and IR64 DRT clustered in the same quadrant perform better for plant height, hundred grain weight, panicle length, flag leaf length, grain length and single plant yield. The genotypes viz., CO51, RNR 15048 and ADT 56 grouped together had high number of productive tillers per plant. The genotypes in the opposite quadrant viz., WGL 21356, CB 19127 and CO 51 Pyr A7 were poor performers for yield attributing traits.

### Molecular Diversity

#### UPGMA Clustering

**Fig. 6:**
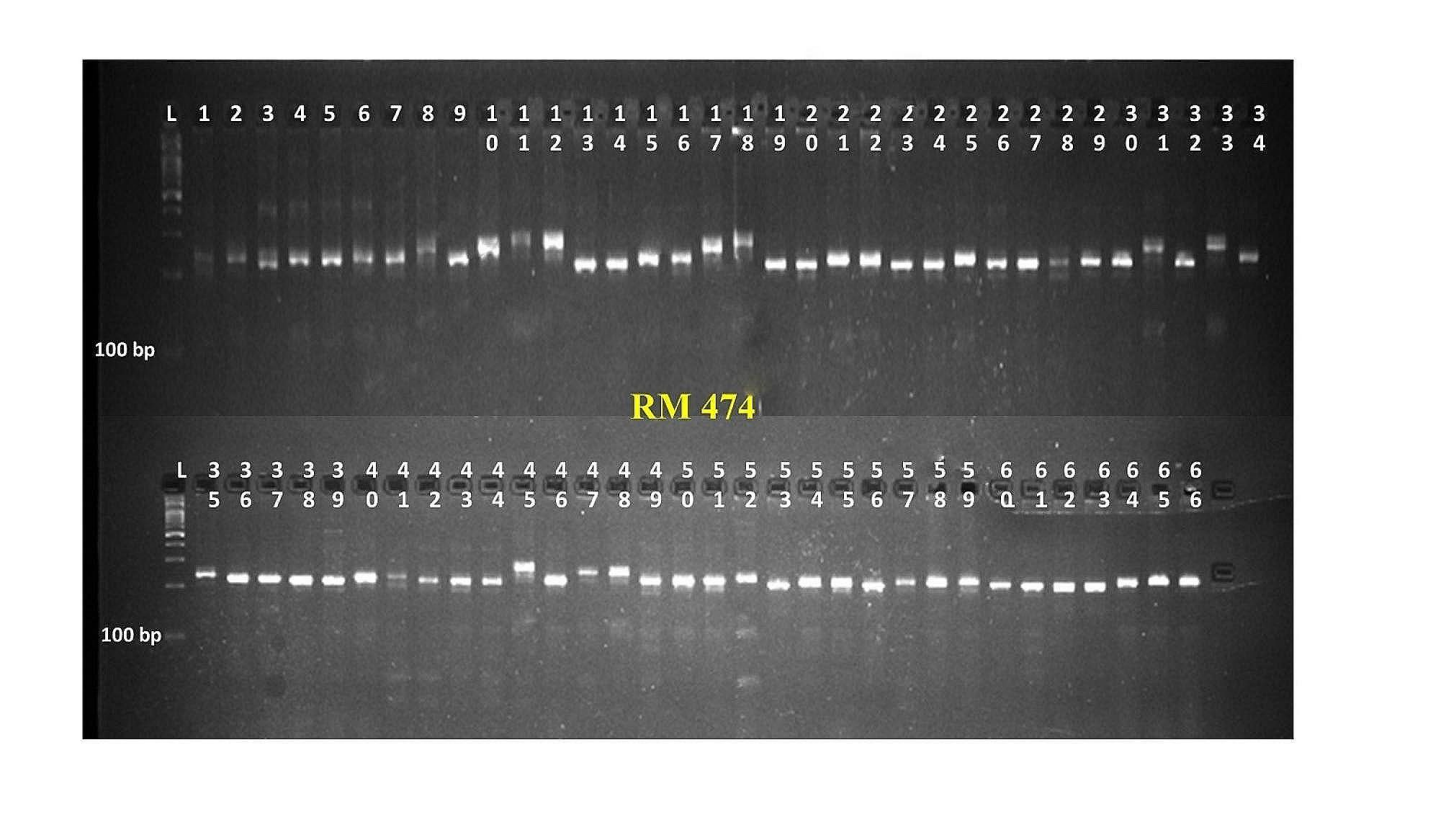
Banding pattern of RM 474

**Fig. 7:**
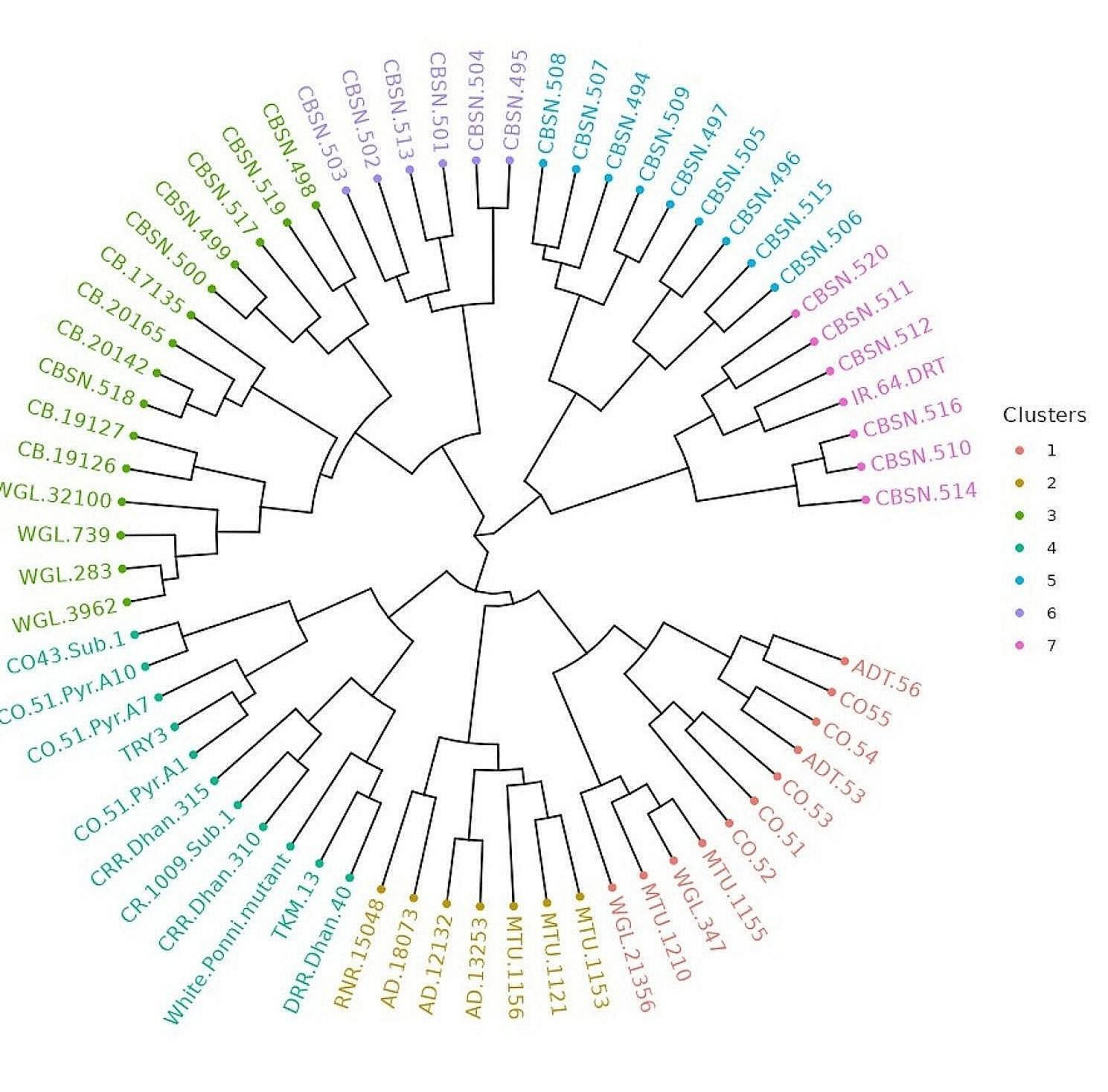
The clustering of parental lines based on jaccard distance

Thirty Polymorphic Simple Sequence Repeat markers produced a total of 122 alleles among 66 parental lines. The number of alleles produced per marker ranged from 2 (RM1, RM443, RM471, RM555, RM205 and RM267) to 9 (RM474) with an average of 4 alleles per marker (Table 8). The marker RM474 (Fig. 6) recorded the highest values for Polymorphic Information Content (PIC) (0.83) and heterozygosity index (0.85). Conversely, RM267 had the lowest PIC (0.28) and heterozygosity index (0.33). The average values for PIC and heterozygosity index were 0.55 and 0.61 respectively. The genotypes were assembled in seven clusters based on Jaccard distances (dissimilarity coefficient) using the Unweighted Pair Group method with Arithmetic Mean (UPGMA) (Table S3 and Fig. 7). The largest cluster with 15 genotypes was cluster IV followed by cluster I and III with 11 genotypes each. The cluster VI contained 9 genotypes, clusters II and VII with 7 genotypes each and the smallest cluster V with 6 genotypes. The maximum Jaccard distance (Jaccard distances are given in Supplementary material S4) was found between CR1009 Sub1 and CBSN 514 (0.95) followed by TRY3 and CBSN 497 (0.93) and between CRR Dhan 310 and CBSN 520(0.93). The genotypes between which minimum Jaccard distance recorded were CBSN 510 and CBSN 516 (0.18) followed by WGL 283 and WGL 3962 (0.20), CO43 Sub 1 and CO51 Pyr A10 (0.21) and CB 20142 and CBSN 518 (0.21).

**Table 8:** The list of polymorphic markers along with their number of alleles, Polymorphic Information Content (PIC) value and heterozygosity index

S.No.	Marker	No of alleles	PIC	H
1	RM1	2	0.35	0.45
2	RM31	5	0.71	0.75
3	RM44	3	0.48	0.54
4	RM205	2	0.36	0.46
5	RM208	4	0.64	0.70
6	RM216	4	0.59	0.66
7	RM228	5	0.61	0.67
8	RM232	6	0.75	0.78
9	RM258	8	0.72	0.75
10	RM267	2	0.28	0.33
11	RM313	3	0.67	0.72
12	RM315	6	0.71	0.75
13	RM443	2	0.33	0.41
14	RM445	6	0.62	0.64
15	RM461	3	0.59	0.66
16	RM471	2	0.36	0.47
17	RM474	9	0.83	0.85
18	RM481	4	0.63	0.69
19	RM514	4	0.49	0.54
20	RM555	2	0.31	0.38
21	RMS-SF21 − 5	5	0.50	0.55
22	RM1108	3	0.58	0.65
23	RM3317	4	0.55	0.63
24	RM3530	6	0.76	0.79
25	RM5359	5	0.69	0.74
26	RM6344	3	0.43	0.51
27	RM6869	3	0.57	0.65
28	RM10318	5	0.47	0.50
29	RMS-PPR9 − 1	3	0.55	0.62
30	DRRMRf3 10	3	0.45	0.53

### Population Structure

**Fig. 8:**
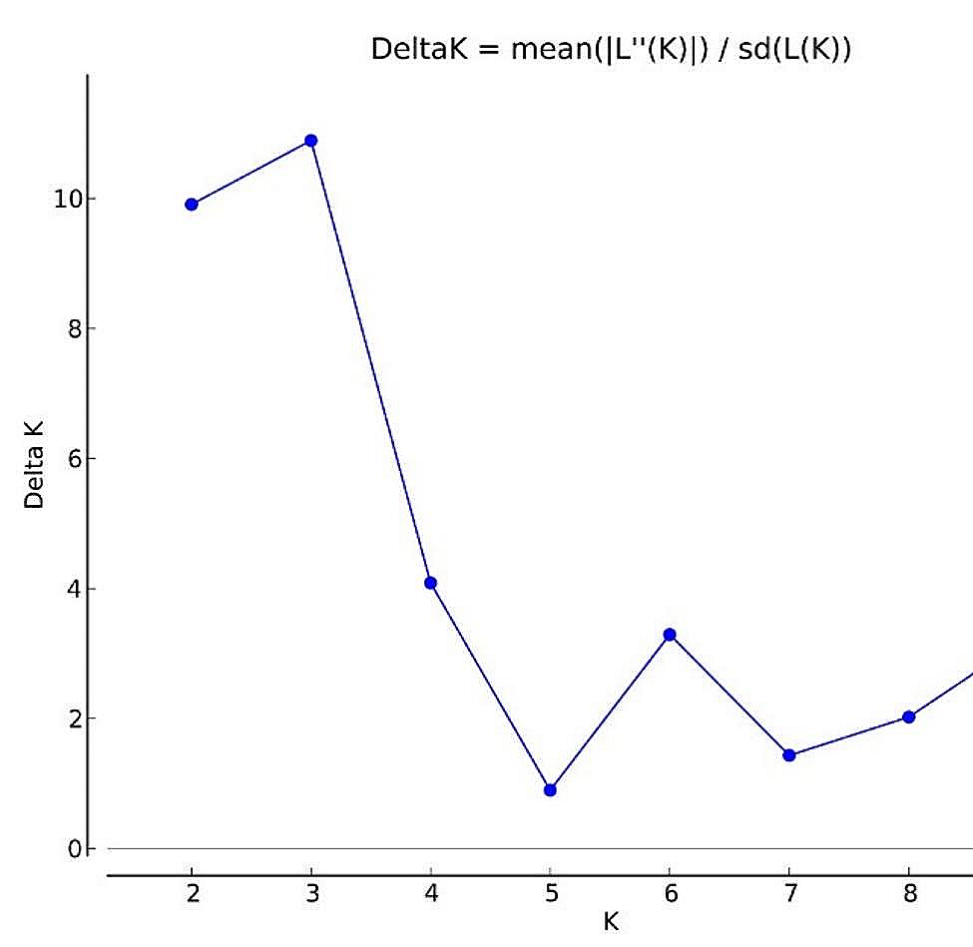
The relation between number of subpopulations and Δ*k*

**Fig. 9:**
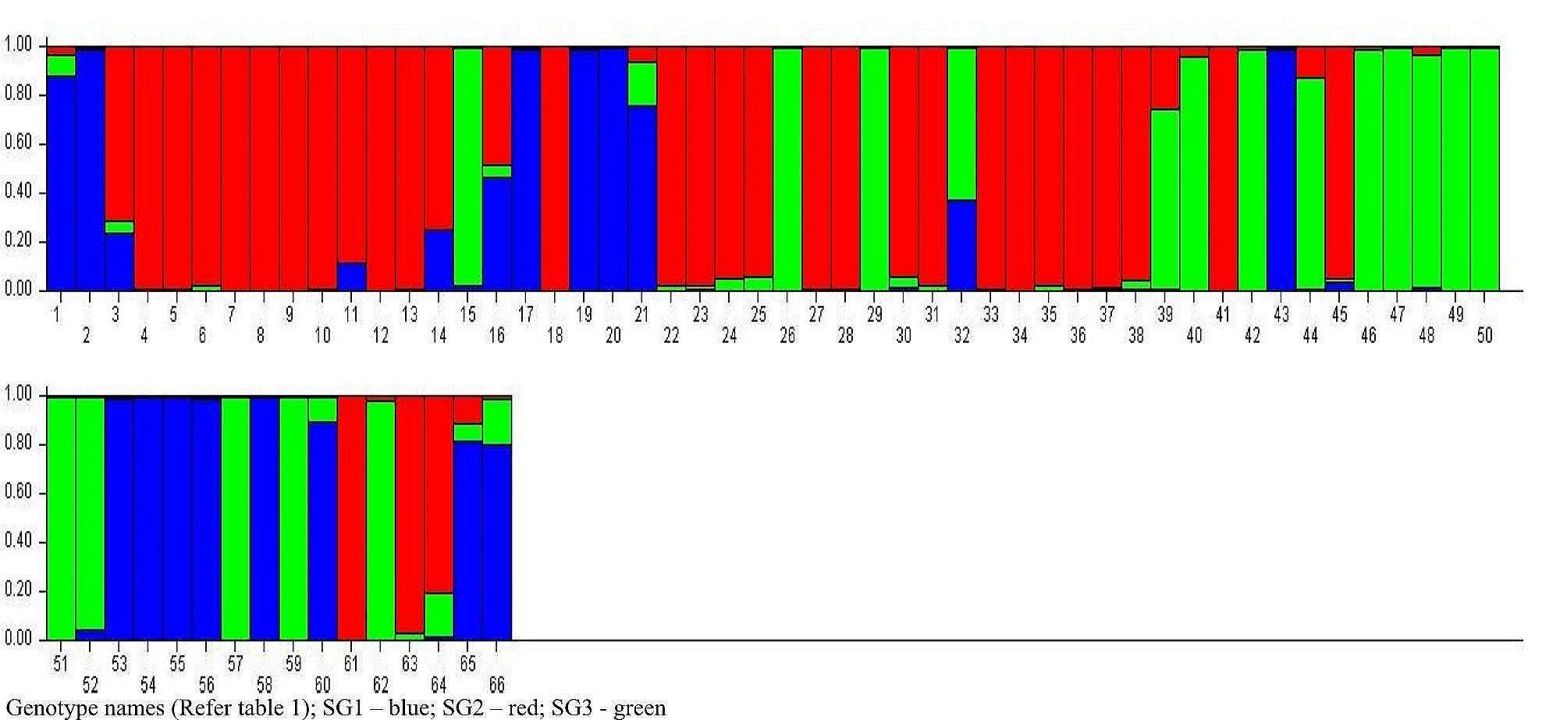
Pictorial representation of distribution of parental lines into different subgroups

The population structure analysis using Bayesian model-based approach in STRUCTURE 2.3.4 software was conducted on 66 genotypes. The program was set at 50,000 burns in iterations with number of subpopulations *k* from 1 to 10. The best *k* value was determined by plotting likelihood value LnP(D) against ad hoc statistics (Δ*k*) according to Evanno et al. (2005). The maximum value for Δ*k* (10.89) was attained when k = 3 (Fig. 8). Therefore, the entire material was divided into 3 subpopulations (Fig. 9). The second subgroup (SG2) was the largest with 32 genotypes (56.25% pure and 43.75% admixture), followed by first subgroup (SG1) with 16 (62.5% pure and 37.5% admixture) and third subgroup (SG3) with 18 genotypes (55.56% pure and 44.44% admixture) respectively. The Analysis of Molecular Variance (AMOVA) delineated the total genetic variation, revealing that 80% of the variation existed among individuals within populations. The genetic divergence among populations accounted for 16%, while variation within individuals contributed 4% to the total variation (Table 9, Supplementary figure 1). On examining pairwise genetic differentiation (Fst) between subpopulations, SG2 and SG3 registered large divergence (0.171) which suggested limited gene flow between them as corroborated by the lowest Nm value of 1.212 (Table 10). The overall average Fst and Nm for the populations were 0.159 and 1.321 respectively. The quantification of genetic diversity parameters identified SG1 and SG2 with highest expected and observed heterozygosity (0.563, 0.036) respectively. SG3 displayed lowest estimates for both expected and observed heterozgosity (0.485, 0.015). The clusters formed in STRUCTURE analysis were not in congruent with biometrical clustering which were evident through the disparity in the number of clusters formed based on molecular and morphological data. In the morphological clusters, there was a co-mingling of genotypes representing both *indica* and *indica-japonica* derivatives. In contrast, the population structure analysis conducted through STRUCTURE distinctly categorized the subspecies, allocating all *indica-japonica* cross derivatives to SG3, while genotypes belonging to the indica subspecies were segregated into SG1 and SG2. Furthermore, the grouping of all improved varieties in a single subgroup (SG2) in STRUCTURE contrasts with their disparate placement across different clusters in the D^2^ grouping. This discrepancy may be attributed to environmental and seasonal influences, which are known to impact morphological clustering.

**Table 9:** Analysis of molecular variance between subpopulations

Source	df	SS	MS	Estimated Variance	Percent variation
Among Populations	2	162.499	81.249	1.564	16%
Among Individuals	63	1011.047	16.048	7.835	80%
Within Individuals	66	25.000	0.379	0.379	4%
Total	131	1198.545		9.777	100%

**Table 10:** Pairwise genetic differentiation (Fst) and gene flow (Nm) between three subpopulations along with observed and expected heterozygosity

	Sub group 1	Sub group 2	Sub group 3		Ho	He
Sub group 1	0.000	1.435	1.315		0.015	0.563
Sub group 2	0.148	0.000	1.212		0.036	0.525
Sub group 3	0.160	0.171	0.000		0.015	0.485

Fst values below diagonal and Nm values above diagonal

## Discussion

In the light of surging global population and depleting resources, it is crucial to explore alternatives that enhance the productivity of rice in major regions worldwide. This necessitates a shift towards leveraging heterosis to overcome the challenges posed by the yield plateau of existing varieties. A deep understanding of the genetic diversity among parental lines is essential for optimizing hybrid breeding outcomes. With this goal, we initiated an investigation involving 66 rice genotypes to assess the genetic landscape and provide insights for the judicious selection of parents in effective hybrid breeding.

The study of genetic parameters revealed that PCV was more than GCV which indicated the influence of environmental parameters other than genes in the determination of phenotype. The number of productive tillers per plant, single plant yield and hundred grain weight had high PCV, GCV, heritability and genetic advance. The traits days to 50% flowering, plant height, grain length and grain length breadth ratio also possessed high heritability, GAM with moderate GCV. These traits offer wide variability coupled with additive gene action and therefore selection based on these traits is appreciable for choosing parents. Similar report for yield and grain characters were furnished by Duraiswamy et al. (2023) and Khalequzzaman et al. (2023) respectively. In D^2^ statistics, the clustering of 66 genotypes in ten different clusters indicated sufficient genetic diversity existing in the experimental material. Most of the released varieties from Tamil Nadu and Andhra Pradesh were grouped together in cluster I while, varieties released from Tamil Nadu were also grouped in cluster II. This clearly indicated that there was no association between genetic diversity and geographical origin. This was in agreement with the findings of Bhargavi et al. (2023), Bhoite et al. (2023) and Srinivas et al. (2023). The distribution of *indica-japonica* cross derived lines in different clusters (III, IV, V, VI and VII) indicated that artificial selection and genetic drift played a significant role in determining genetic diversity. The grouping of genotypes in cluster V with less intra cluster distance showed close resemblance for days to 50% flowering and could be due to unidirectional selection pressure during development of these genotypes (Srinivas et al. 2023). The maximum intra cluster distance for cluster VII and X shall be explained to be the result of past selection history, degree of combining ability, genetic architecture or the genotype heterogeneity (Dinesh et al. 2023). The maximum contribution of days to 50% flowering, hundred seed weight and plant height to the genetic divergence makes these traits to be the direct selection indices in parental lines. Srinivas et al. (2023), jebakani et al. (2023) and Jangala et al. (2022) also reported 50% flowering, hundred seed weight and plant height with maximum contribution towards divergence. Based on the cluster mean values, the genotypes in cluster VII (Blue Bonnet/CB 87R 5-3-1, CB 17135, CO 51 Pyr A10, CBSN 500) can be used as donors for producing hybrids with high yield and high panicle length. The genotypes in cluster III (CBSN 499, WGL 347 and WGL 283) can be employed as donors to produce dwarf and early maturing hybrids. The *indica-japonica* cross derivative CBSN 495 in the solitary cluster shall be employed as a parent in three-line breeding in order to achieve high yield with good restorability of fertile hybrids. The higher inter cluster distances than intra cluster distances express enough genetic variability to be present among the genotypes (Jebakani et al. 2023). On combining high inter cluster distance and cluster mean, the crosses between genotypes in clusters III, IX and cluster V, cluster VII and VI are beneficial to obtain superior hybrids.

Principal component analysis (PCA) measures the spatial distance between genotypes, contrasting with D^2^ statistics (Nadarajan et al. 2016). The contribution of days to 50% flowering, plant height, panicle length, flag leaf length, hundred grain weight, grain length and grain breadth for PC1 in our study is supported by the reports of Nachimuthu et al. (2014), Kathare et al. (2023), Duraiswamy et al. (2023) and Gupte et al. (2023). The traits occurring together in different principal components with maximum positive contribution viz., days to 50% flowering, hundred grain weight and grain breadth tend to remain together and so prior importance should be given for positive selection of them in breeding programme (Kumari et al. 2023). In PCA biplot, the genotypes viz., CBSN 495, CB 17135, CBSN 494, CRR Dhan 315, CRR Dhan 310 and IR64 DRT which are good performers for yield contributing traits were positioned around the corresponding trait vectors in the same quadrant. The genotype WGL 347, despite high yield appeared in a different quadrant due to low plant height, making it a viable parent. All these genotypes identified in PCA were assembled in the diverse clusters (III, V, VI, VII and IX) by D^2^ statistics.

The cluster analysis by Jaccard distance for SSR scoring grouped the 66 parental lines into seven clusters. The released varieties from Tamil Nadu, Andhra Pradesh and Telangana were grouped in cluster I and II reflecting similarity in parentage for medium slender grain types preferred in those regions. Bhattacharjee et al. (2021) also reported marker-based grouping independent of geographical origin. The average PIC (0.55) and the number of alleles (4) were in close accordance with the reports of Tripathi et al. (2020) who reported average alleles of 3.7 and average PIC of 0.56 in molecular diversity analysis of 27 rice cultivars using 12 SSR markers and Salem and Sallam (2016) who obtained average values of 4.5 and 0.57 for number of alleles per locus and PIC respectively in genetic diversity study of 22 Egyptian and exotic rice genotypes using 23 SSR markers. Whereas Pandita et al. (2023), Akter et al. (2022) and Bajracharya et al. (2006) reported lower mean values than our findings for PIC and number of alleles per locus. The most informative marker with the highest PIC value was identified to be RM474. Based on Jaccard distance, the minimum genetic diversity was found among released varieties which shall be attributed to their free gene flow and shared genetic architecture. Among PCA identified good performing genotypes, high dissimilarity coefficients of 0.90 between CRR Dhan 315 and CBSN 494, 0.83 between CRR Dhan 310 and IR64 DRT and 0.85 between CBSN 495 and CB 17135 were registered. These genotypes also belonged to different molecular clusters reinforcing their genetic distinctiveness. In population structure analysis, the best *k* value was identified to be 3 which was in accordance with the results of Mishra et al. (2019) in 35 germplasm accessions, Nachimuthu et al. (2015) in 192 rice germplasm lines and Upadhyay et al. (2012) in 25 rice varieties. Most of the genotypes with admixtures were identified to be wild rice derivatives and *indica-japonica* cross derivatives. The admixtures may be due to the constitution of inherited alleles as a result of artificial crossing and hybridization (Yamasaki and Ideta 2013). The analysis of molecular variance identified significant genetic variation of 80% attributed to differences among individuals within the population. This highlighted the presence of ample variation in the genotypes that can be harnessed in breeding programmes. The pairwise Fst values exceeding 0.15 as propounded by Wright (1978) indicated large genetic differentiation. The lower genetic differentiation and high gene flow observed between SG1 and SG2 was postulated to be the consequence of shared evolutionary history of common parents for released and improved varieties, all belonging to *indica* subspecies and the exchange of genetic materials across states for breeding. In contrast, SG3 encompassing genotypes from *indica-japonica* cross derivatives, exhibited a noteworthy and large genetic differentiation from other two subpopulations. The average observed heterozygosity was 0.022 which was lower than the reports of Tarang et al. (2016) and Suvi et al. (2020). This low estimate can be ascertained to the autogamous mode of reproduction in rice. On the other hand, the average expected heterozygosity, estimated at 0.524, aligned with the findings of Nachimuthu et al. (2015). This can be attributed to the exchange of genes among the genotypes resulting in broad spectrum of genetic diversity. Out of 3 subpopulations, SG3 distinctly encompassed all the *indica-japonica* cross derivatives in which *indica* lines were confirmed for the presence of restorer genes. This subgroup could be exploited to select good restorers based on mean value for yield attributes.

The comparison of morphological and molecular cluster revealed that the number of clusters and distribution of genotypes in clusters were different in both approaches. This swapping of genotypes might be attributed to the influence of environment and genotype-environment interaction in determining the morphology. Similar pattern of differences in clusters of morphological and molecular marker analysis were reported by Pathak et al. (2020), Rahman et al. (2011), Vengadessan et al. (2016) and Han-Yong et al. (2004). But the good performing genotypes identified through PCA were positioned in different clusters in both clustering which enable us to select diverse parents for producing superior hybrids.

## Conclusion

The present experimental material exhibited a wide genetic divergence in both D^2^ statistics and Jaccard distance-based analyses. Two lines viz., CBSN 495 and CBSN 494 located in different clusters were identified as the potential donors for short duration hybrids. The cross between WGL 347, CB 17135 and improved varieties viz., CRR Dhan 310 CRR Dhan 315, IR64 DRT assembled in different clusters shall be attempted to develop nutritionally improved and drought tolerant hybrids. Henceforth, a more precise parental selection for hybridization programme is possible by collaborative understanding of morphological and molecular clusters.

### Electronic Supplementary Material

Below is the link to the electronic supplementary material.

Supplementary Material 1

Supplementary Material 2

Supplementary Material 3

## Data Availability

No datasets were generated or analysed during the current study.
