# Supplementary material for: Assessing the Genetic Diversity of Parents for Developing Hybrids Through Morphological and Molecular Markers in Rice (Oryza sativa L.)
Source: Rice (N Y). 2024 Feb 24;17:17. doi: 10.1186/s12284-024-00691-2 (PMC10894128; doi:10.1186/s12284-024-00691-2)

**Table S1.** **The mean values of genotypes for all the biometrical traits**

| **S. No.** | **Genotypes** | **DFF** | **PH** | **NPTP** | **PL** | **FLL** | **SPY** | **HGW** | **GL** | **GB** | **L/B** |
| --- | --- | --- | --- | --- | --- | --- | --- | --- | --- | --- | --- |
| 1 | CO 51 | 94 | 65.00 | 27 | 18.00 | 25.00 | 24.67 | 1.50 | 0.78 | 0.20 | 3.90 |
| 2 | AD 12132 | 95 | 58.33 | 14 | 21.50 | 29.80 | 28.75 | 1.36 | 0.78 | 0.24 | 3.25 |
| 3 | CO 52 | 103 | 77.33 | 29 | 28.00 | 32.33 | 18.50 | 1.56 | 0.75 | 0.24 | 3.13 |
| 4 | CO55 | 107 | 88.00 | 24 | 19.00 | 29.20 | 25.33 | 1.65 | 0.80 | 0.23 | 3.48 |
| 5 | ADT 53 | 93 | 62.00 | 18 | 18.00 | 26.00 | 25.50 | 1.77 | 0.70 | 0.22 | 3.18 |
| 6 | WGL 283 | 76 | 83.00 | 15 | 23.00 | 32.33 | 10.00 | 1.84 | 0.92 | 0.26 | 3.54 |
| 7 | ADT 56 | 91 | 73.00 | 23 | 20.00 | 28.33 | 25.50 | 1.37 | 0.90 | 0.24 | 3.75 |
| 8 | TRY3 | 97 | 81.33 | 13 | 23.00 | 24.47 | 11.00 | 1.70 | 0.80 | 0.30 | 2.67 |
| 9 | CO43 Sub 1 | 114 | 86.00 | 23 | 23.00 | 30.53 | 34.50 | 1.70 | 0.78 | 0.28 | 2.79 |
| 10 | CR 1009 Sub 1 | 115 | 74.00 | 17 | 21.00 | 26.67 | 22.00 | 1.60 | 0.72 | 0.32 | 2.25 |
| 11 | CO 54 | 107 | 68.00 | 12 | 24.00 | 29.00 | 25.00 | 1.36 | 0.72 | 0.20 | 3.60 |
| 12 | CRR Dhan 315 | 105 | 108.67 | 16 | 24.00 | 34.73 | 44.33 | 2.13 | 0.88 | 0.28 | 3.14 |
| 13 | TKM 13 | 95 | 66.67 | 19 | 24.00 | 31.40 | 16.00 | 1.50 | 0.74 | 0.24 | 3.08 |
| 14 | White Ponni mutant | 92 | 59.67 | 23 | 20.00 | 30.67 | 22.00 | 1.27 | 0.72 | 0.22 | 3.27 |
| 15 | CBSN 494 | 90 | 123.00 | 17 | 32.00 | 29.67 | 42.00 | 1.67 | 0.87 | 0.23 | 3.78 |
| 16 | RNR 15048 | 100 | 75.67 | 30 | 26.00 | 31.00 | 26.50 | 1.09 | 0.78 | 0.20 | 3.90 |
| 17 | AD 18073 | 117 | 59.67 | 15 | 16.00 | 31.67 | 16.00 | 1.43 | 0.66 | 0.30 | 2.20 |
| 18 | CRR Dhan 310 | 96 | 107.67 | 22 | 26.00 | 36.07 | 37.00 | 1.94 | 0.86 | 0.28 | 3.07 |
| 19 | AD 13253 | 98 | 90.00 | 15 | 25.00 | 37.37 | 33.00 | 1.73 | 0.78 | 0.28 | 2.79 |
| 20 | MTU 1121 | 93 | 71.67 | 18 | 20.00 | 32.00 | 24.00 | 1.87 | 0.76 | 0.26 | 2.92 |
| 21 | MTU 1156 | 90 | 71.67 | 19 | 27.00 | 29.00 | 26.50 | 2.27 | 0.80 | 0.28 | 2.86 |
| 22 | DRR Dhan 40 | 96 | 75.67 | 22 | 23.60 | 35.83 | 35.00 | 1.35 | 0.76 | 0.28 | 2.71 |
| 23 | MTU 1210 | 91 | 71.00 | 17 | 20.00 | 31.67 | 23.00 | 1.44 | 0.74 | 0.26 | 2.85 |
| 24 | WGL 347 | 78 | 70.33 | 18 | 19.00 | 26.67 | 36.50 | 1.52 | 0.78 | 0.24 | 3.25 |
| 25 | WGL 21356 | 81 | 68.33 | 21 | 19.00 | 27.33 | 7.50 | 0.93 | 0.70 | 0.18 | 3.89 |
| 26 | CBSN 495 | 85 | 118.33 | 12 | 24.00 | 26.33 | 9.50 | 2.57 | 0.92 | 0.32 | 2.88 |
| 27 | WGL 739 | 94 | 78.67 | 25 | 20.00 | 29.00 | 14.50 | 2.10 | 0.82 | 0.26 | 3.15 |
| 28 | WGL 3962 | 84 | 74.33 | 14 | 19.00 | 30.00 | 18.50 | 2.36 | 0.86 | 0.24 | 3.58 |
| 29 | CBSN 496 | 90 | 95.33 | 19 | 27.00 | 29.00 | 24.00 | 1.76 | 0.82 | 0.28 | 2.93 |
| 30 | WGL 32100 | 90 | 67.33 | 22 | 21.00 | 31.67 | 25.00 | 1.32 | 0.76 | 0.22 | 3.45 |
| 31 | CB 19127 | 96 | 77.00 | 14 | 19.00 | 22.67 | 20.50 | 1.13 | 0.66 | 0.26 | 2.54 |
| 32 | CBSN 497 | 113 | 88.00 | 21 | 22.00 | 26.67 | 8.50 | 1.96 | 0.80 | 0.24 | 3.33 |
| 33 | CB 17135 | 83 | 110.00 | 10 | 24.00 | 30.00 | 35.50 | 1.49 | 0.80 | 0.24 | 3.33 |
| 34 | CB 20165 | 99 | 74.67 | 16 | 18.00 | 24.00 | 21.50 | 1.32 | 0.76 | 0.20 | 3.80 |
| 35 | CB 20142 | 87 | 69.00 | 12 | 18.00 | 26.33 | 29.00 | 1.43 | 0.78 | 0.24 | 3.25 |
| 36 | CO 51 Pyr A10 | 86 | 77.00 | 15 | 19.00 | 15.00 | 22.00 | 1.54 | 0.76 | 0.26 | 2.92 |
| 37 | CBSN 498 | 80 | 64.33 | 19 | 24.00 | 25.00 | 12.00 | 2.09 | 0.86 | 0.24 | 3.58 |
| 38 | MTU 1155 | 84 | 78.67 | 22 | 19.00 | 21.00 | 25.50 | 1.92 | 0.76 | 0.30 | 2.53 |
| 39 | CBSN 499 | 75 | 56.33 | 16 | 20.00 | 24.33 | 17.50 | 2.01 | 0.90 | 0.28 | 3.21 |
| 40 | CBSN 500 | 87 | 84.67 | 28 | 27.00 | 26.67 | 15.00 | 1.92 | 0.80 | 0.26 | 3.08 |
| 41 | CO 51 Pyr A1 | 85 | 65.00 | 16 | 19.00 | 22.33 | 7.00 | 1.23 | 0.76 | 0.26 | 2.92 |
| 42 | CBSN 501 | 109 | 86.67 | 21 | 23.00 | 30.00 | 7.00 | 1.94 | 0.86 | 0.24 | 3.58 |
| 43 | MTU 1153 | 90 | 75.00 | 19 | 23.00 | 31.00 | 27.00 | 2.02 | 0.80 | 0.26 | 3.08 |
| 44 | CBSN 502 | 118 | 104.00 | 14 | 23.00 | 28.67 | 10.50 | 1.76 | 0.74 | 0.26 | 2.85 |
| 45 | CB 19126 | 96 | 73.00 | 13 | 21.00 | 28.00 | 18.00 | 1.23 | 0.72 | 0.24 | 3.00 |
| 46 | CBSN 503 | 116 | 83.33 | 15 | 28.00 | 30.33 | 14.00 | 1.76 | 0.76 | 0.30 | 2.53 |
| 47 | CBSN 504 | 92 | 92.67 | 13 | 23.00 | 32.00 | 11.00 | 1.52 | 0.80 | 0.26 | 3.08 |
| 48 | CBSN 505 | 118 | 72.67 | 14 | 22.00 | 32.00 | 6.00 | 1.57 | 0.80 | 0.28 | 2.86 |
| 49 | CBSN 506 | 92 | 94.67 | 15 | 24.00 | 29.67 | 16.50 | 1.43 | 0.76 | 0.28 | 2.71 |
| 50 | CBSN 507 | 89 | 106.00 | 14 | 25.00 | 29.67 | 12.00 | 2.00 | 0.81 | 0.26 | 3.12 |
| 51 | CBSN 508 | 93 | 70.33 | 28 | 23.00 | 27.33 | 14.50 | 1.99 | 0.88 | 0.24 | 3.67 |
| 52 | CBSN 509 | 120 | 82.67 | 21 | 24.00 | 29.33 | 17.50 | 2.12 | 0.82 | 0.24 | 3.42 |
| 53 | CBSN 510 | 95 | 116.00 | 12 | 26.00 | 32.00 | 11.00 | 2.38 | 0.78 | 0.34 | 2.29 |
| 54 | CBSN 511 | 126 | 88.00 | 18 | 21.00 | 23.00 | 14.00 | 1.70 | 0.76 | 0.26 | 2.92 |
| 55 | IR 64 DRT | 113 | 68.67 | 17 | 19.00 | 20.00 | 34.00 | 2.30 | 0.96 | 0.30 | 3.20 |
| 56 | CBSN 512 | 125 | 119.33 | 18 | 22.00 | 31.33 | 12.00 | 2.51 | 0.78 | 0.30 | 2.60 |
| 57 | CBSN 513 | 102 | 88.33 | 24 | 28.00 | 28.67 | 12.00 | 1.80 | 0.82 | 0.24 | 3.42 |
| 58 | CBSN 514 | 100 | 92.67 | 21 | 26.00 | 28.33 | 9.50 | 2.21 | 0.76 | 0.32 | 2.38 |
| 59 | CBSN 515 | 91 | 84.00 | 11 | 23.00 | 32.67 | 14.50 | 1.25 | 0.77 | 0.29 | 2.66 |
| 60 | CBSN 516 | 125 | 85.33 | 19 | 19.00 | 37.67 | 15.00 | 2.07 | 0.74 | 0.32 | 2.31 |
| 61 | CO 51 Pyr A7 | 86 | 63.67 | 15 | 18.00 | 20.83 | 14.00 | 1.27 | 0.76 | 0.24 | 3.17 |
| 62 | CBSN 517 | 88 | 100.00 | 21 | 22.00 | 24.67 | 15.00 | 1.75 | 0.84 | 0.24 | 3.50 |
| 63 | CBSN 518 | 82 | 61.00 | 14 | 24.00 | 22.33 | 12.50 | 1.96 | 0.88 | 0.28 | 3.14 |
| 64 | CBSN 519 | 78 | 70.33 | 25 | 22.00 | 29.67 | 17.50 | 1.91 | 0.92 | 0.30 | 3.07 |
| 65 | CO 53 | 98 | 70.67 | 23 | 23.00 | 30.33 | 18.50 | 2.27 | 0.74 | 0.28 | 2.64 |
| 66 | CBSN 520 | 123 | 94.67 | 16 | 23.00 | 26.00 | 11.50 | 2.00 | 0.84 | 0.20 | 4.20 |
| MIN | | 75 | 56.00 | 10 | 16.00 | 15.00 | 6.00 | 0.93 | 0.66 | 0.18 | 2.20 |
| MAX | | 126 | 123.00 | 30 | 32.00 | 37.67 | 44.33 | 2.57 | 0.96 | 0.34 | 4.20 |
| SD | | 13.13 | 16.18 | 4.74 | 3.14 | 4.18 | 9.133 | 0.37 | 0.06 | 0.03 | 0.45 |

DFF – Days to 50% flowering; PH – Plant height; NPTP – Number of productive tillers per plant; PL – Panicle length; FLL – Flag leaf length; SPY – Single plant yield; HGW – Hundred grain weight; GL – Grain length; GB – Grain breadth; L/B – Grain Length Breadth ratio

**Table S2.** **The contribution of each trait to the total divergence**

| **Trait** | **No of times 1st rank** | **Contribution** |
| --- | --- | --- |
| DFF | 1708 | 79.63 |
| PH | 111 | 5.17 |
| NPTP | 0 | 0.00 |
| PL | 59 | 2.75 |
| FLL | 1 | 4.66 |
| SPY | 29 | 1.35 |
| HGW | 167 | 7.79 |
| GL | 5 | 0.23 |
| GB | 17 | 0.79 |
| L/B | 48 | 2.24 |
| Total | 2145 |  |

**Table S3.** **The clustering of genotypes based on Jaccard distance**

| **Cluster** | **No of genotypes** | **Name of genotypes** |
| --- | --- | --- |
| I | 11 | ADT 56, CO55, CO 54, ADT 53, CO 51, CO 52, CO 53, MTU 1210, WGL 347, WGL 21356, MTU 1155 |
| II | 7 | AD 12132, RNR 15048, AD 18073, AD 13253, MTU 1121, MTU 1156, MTU 1153 |
| III | 11 | TRY3, CO43 Sub 1, CR 1009 Sub 1, CRR Dhan 315, TKM 13, White Ponni mutant, CRR Dhan 310, DRR Dhan 40, CO 51 Pyr A10, CO 51 Pyr A1, CO 51 Pyr A7 |
| IV | 15 | WGL 3962, WGL 283, WGL 739, WGL 32100, CB 19127, CB 19126, CB 20165, CB 20142, CB 17135, CB 19126, CBSN 499, CBSN 500, CBSN 518, CBSN 519, CBSN 517 |
| V | 6 | CBSN 495, CBSN 501, CBSN 502, CBSN 503, CBSN 504, CBSN 513, |
| VI | 9 | CBSN 495, CBSN 496, CBSN 497, CBSN 505, CBSN 506, CBSN 507, CBSN 508, CBSN 509, CBSN 515 |
| VII | 7 | CBSN 510, CBSN 511, IR 64 Drought, CBSN 512, CBSN 514, CBSN 516, CBSN 520 |

**Supplementary figure 1. The percentage of molecular variance identified through AMOVA**


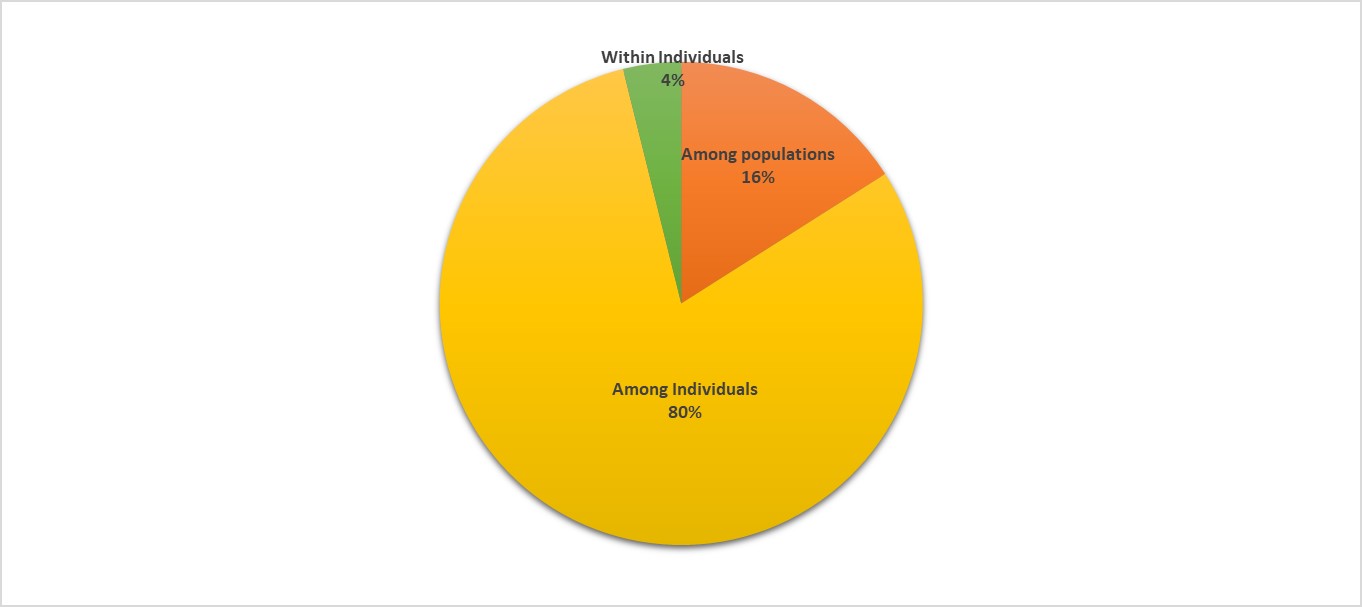

Supplement: Supplementary file 1 — Supplementary Material 1 [file 12284_2024_691_MOESM1_ESM.docx]
