# Supplementary figures and images for: Assessing the Genetic Diversity of Parents for Developing Hybrids Through Morphological and Molecular Markers in Rice (Oryza sativa L.)
Source: Rice (N Y). 2024 Feb 24;17:17. doi: 10.1186/s12284-024-00691-2 (PMC10894128; doi:10.1186/s12284-024-00691-2)

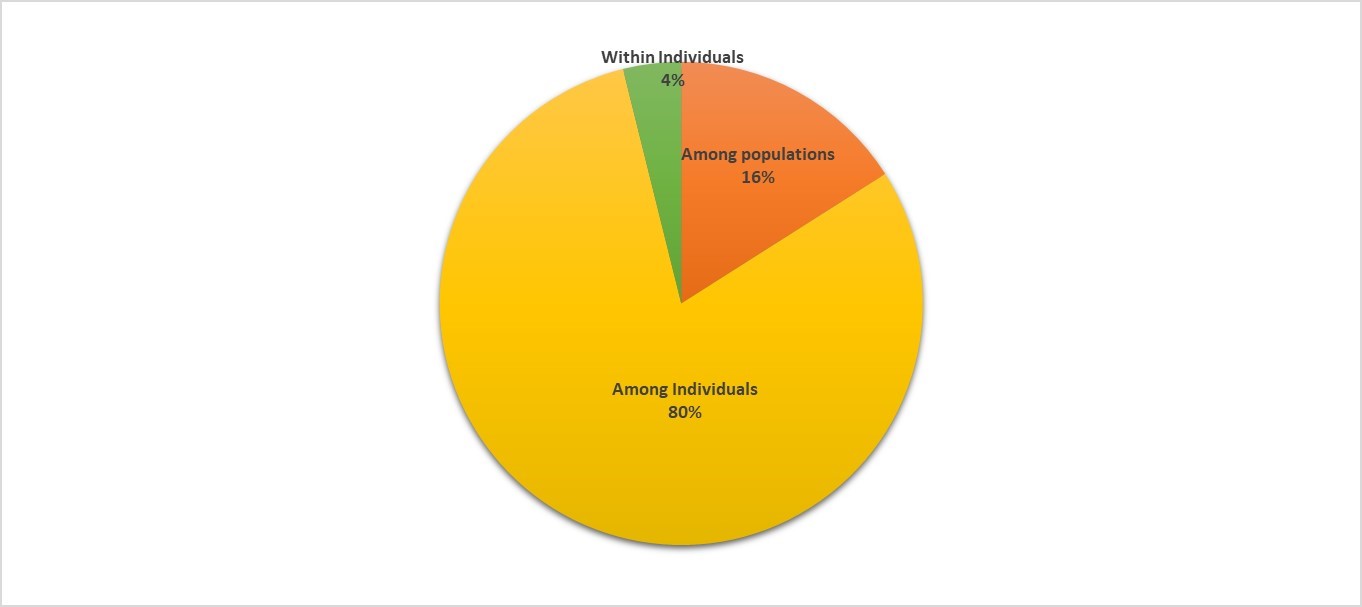

Supplement: Supplementary file 3 — Supplementary Material 3 [file 12284_2024_691_MOESM3_ESM.jpg]
